# Marburg virus regulates the IRE1/XBP1-dependent unfolded protein response to ensure efficient viral replication

**DOI:** 10.1080/22221751.2019.1659552

**Published:** 2019-09-07

**Authors:** Cornelius Rohde, Stephan Becker, Verena Krähling

**Affiliations:** a Institut für Virologie, Philipps-Universität Marburg, Marburg, Germany; b Deutsches Zentrum für Infektionsforschung (DZIF), Gießen – Marburg – Langen, Marburg, Germany

**Keywords:** Marburg virus, VP30, GP, ER stress, IRE1, XBP1

## Abstract

Viruses regulate cellular signalling pathways to ensure optimal viral replication. During Marburg virus (MARV) infection, large quantities of the viral glycoprotein GP are produced in the ER; this may result in the activation of the unfolded protein response (UPR). The most conserved pathway to trigger UPR is initiated by IRE1. Activation of IRE1 results in auto-phosphorylation, splicing of the *XBP1* mRNA and translation of the XBP1s protein. XBP1s binds cis-acting UPR elements (UPRE) which leads to the enhanced expression of genes which should restore ER homeostasis. XBP1u protein is translated, if IRE1 is not activated. Here we show that ectopic expression of MARV GP activated the IRE1-XBP1 axis of UPR as monitored by UPRE luciferase assays. However, while at 24 h of infection with MARV IRE1 was phosphorylated, expression of XBP1s was only slightly enhanced and UPRE activity was not detected. The IRE1-XBP1 axis was not active at 48 h p.i. Co-expression studies of MARV proteins demonstrated that the MARV protein VP30 suppressed UPRE activation. Co-immunoprecipitation analyses revealed an RNA-dependent interaction of VP30 with XBP1u. Knock-out of IRE1 supported MARV infection at late time points. Taken together, these results suggest that efficient MARV propagation requires specific regulation of IRE1 activity.

## Introduction

Viral infections impose stress on infected host cells. To counteract cellular stress responses and to ensure efficient viral propagation, viruses manipulate cellular signalling pathways [1,2]. During infection with viruses, accumulation of newly synthesized viral glycoproteins in the endoplasmic reticulum (ER) may lead to exhaustion of the folding capacity of the ER, resulting in the activation of the unfolded protein response (UPR) [3]. The UPR serves to maintain ER homeostasis by enhancing the folding capacity of the ER and decreasing the rate of synthesis of new proteins. Prolonged ER stress can trigger terminal UPR, resulting in apoptosis [4,5].

The UPR encompasses three signalling pathways [3,6] that are regulated by sensors of protein folding. These sensors are PKR-like ER kinase (PERK), activating transcription factor 6 (ATF6) and inositol-requiring enzyme 1 (IRE1). Following activation, PERK phosphorylates eukaryotic translation initiation factor 2α (eIF2α) and thereby diminishes translation [3]. Activation of ATF6 and IRE1 induces the differential expression of a whole set of genes whose protein products act to restore the homeostasis of the ER [7].

The most conserved UPR pathway is executed by IRE1, a kinase and endoribonuclease, which mediates the unconventional splicing of *X-box binding protein 1* (*XBP1*) mRNA [3]. The excision of a 26-nucleotide fragment creates an mRNA variant encoding a spliced XBP1 protein, XBP1s, which transcriptionally activates genes controlled by specific promoter sequences, for example, the unfolded protein response element (UPRE) [8]. Activation of UPREs was originally thought to depend solely on XBP1s [9]; however, it was recently found that both ATF6 and XBP1 contribute to the activation of UPRE [7]. Under non-UPR conditions, translation of *XBP1u* mRNA produces “unspliced” XBP1 (XBP1u). A translation-pausing motif in the *XBP1u* mRNA causes a transient pause in XBP1u translation, and the complex of the XBP1u’s nascent protein chain, its own mRNA and the ribosome are recognized by the signal recognition particle and recruited to the ER membrane, where phosphorylated IRE1 mediates splicing of the *XBP1u* mRNA [10].

It has been reported that RNA virus infections, e.g. infection by West Nile virus (WNV), Dengue virus and influenza virus, activate UPR processes [11–13]. Furthermore, it was shown that activated IRE1-dependent signalling may be beneficial or detrimental to viral propagation, and viruses have developed different strategies for coping with and even taking advantage of the ER stress response [13,14].

Marburg virus (MARV) causes outbreaks of severe, often fatal hemorrhagic fever in Central and East Africa [15]. MARV particles are composed of seven viral proteins. The single-stranded negative-sense viral RNA is complexed with the nucleoprotein NP (ribonucleoprotein complex, RNP). In MARV-infected cells, NP induces the formation of viral inclusion bodies, which are found in close association with the rough ER [16] and represent the sites of viral replication and transcription [17]. Associated with the RNP are the viral proteins L, VP35, VP24 and the viral transcription factor VP30 [18,19]. The MARV matrix protein VP40 is the driving force for viral budding [20,21]. GP is synthesized at the ER and further transported to the plasma membrane via the classical secretory pathway [22]. GP is highly glycosylated with mannose-rich and complex-type N-glycans and with mucin-type O-glycans. The majority of both the N- and O-glycans are attached to a mucin-like domain (MLD) [23]. GP plays an essential role during MARV infection of target cells by binding to the cellular receptor and mediating fusion of the viral and cellular membranes. MARV infection results in the production of large amounts of viral proteins in the cytosol. GP is translocated into the ER, where it accumulates; it is only slowly released to the Golgi and transported to the plasma membrane [22].

In recent studies, IRE1 signalling was shown to be a double-edged sword for viral replication, as it can either be pro- or antiviral [13,14]. It is currently unknown how MARV infection influences and, in turn, is influenced by IRE1; it is therefore of interest to investigate whether the accumulation of GP in the ER induces the IRE1-dependent UPR and whether this has implications for viral propagation.

In the present study, we showed that ectopic expression of MARV GP induces XBP1s expression and subsequent UPRE activation. The GP-induced UPRE activity is counteracted during MARV infection. This is probably caused by the MARV transcription factor VP30 which was shown to inhibit UPRE activity most likely by associating with XBP1u.

## Materials and methods

### Cell culture and virus infection

Vero C1008 (ATCC CRL-1586) and HuH7 cells (fully matching the STR reference profile of HuH-7) were cultured in Dulbecco’s modified Eagle’s medium (DMEM) supplemented with 10% foetal calf serum (FCS), penicillin (50 units/mL), streptomycin (50 µg/mL) (P/S) and glutamine (2 mM) (Q). HAP1 parental (Horizon Discovery, Catalog ID: C631) and HAP1 IRE1 knockout (Horizon Discovery, Catalog ID: HZGHC000742c006) cells were cultured in Iscove's Modified Dulbecco's Medium (IMDM) supplemented with 10% FCS, P/S. The Musoke strain of MARV (GenBank accession number NC_001608.03) was propagated in Vero C1008 cells. Virus titre was determined by immunoplaque titration. TCID_50_/ml analyses were conducted as described earlier [24]. All work with filoviruses was performed in the biosafety level 4 (BSL4) facility at the Philipps University of Marburg.

### Molecular cloning

The molecular cloning of plasmids encoding the MARV Musoke-derived proteins NP, HA-NP, GP, VP24, VP30, VP35, VP40, and L and the deletion mutant of GP have been described elsewhere [25–27]. The XBP1 coding sequence derived from HuH7 cells was cloned adding an N-terminal Flag-tag and a C-terminal GFP into the pCAGGS vector (see Figure S4a). hIRE1 wt was a gift from Fumihiko Urano (Addgene plasmid #20744) [28]. The IRE1 coding sequence was subcloned into the pCAGGS vector. MARV Musoke GP-HA was cloned based on pCAGGS-MARV-Musoke-GP [29] by PCR using specific oligonucleotides to add an HA-tag after nucleotide 6801 (reference sequence NC_001608). A hemagglutinin (HA)-tag was joined N-terminally to MARV Musoke VP30 and VP35. The precise cloning strategies are available upon request. Sequencing analysis revealed the correct products and IFA of HA-tagged viral proteins and their respective wild-type counterparts indicated no differences in their localization patterns (author’s observation, not published).

### Luciferase reporter assays

**Figure 1.**
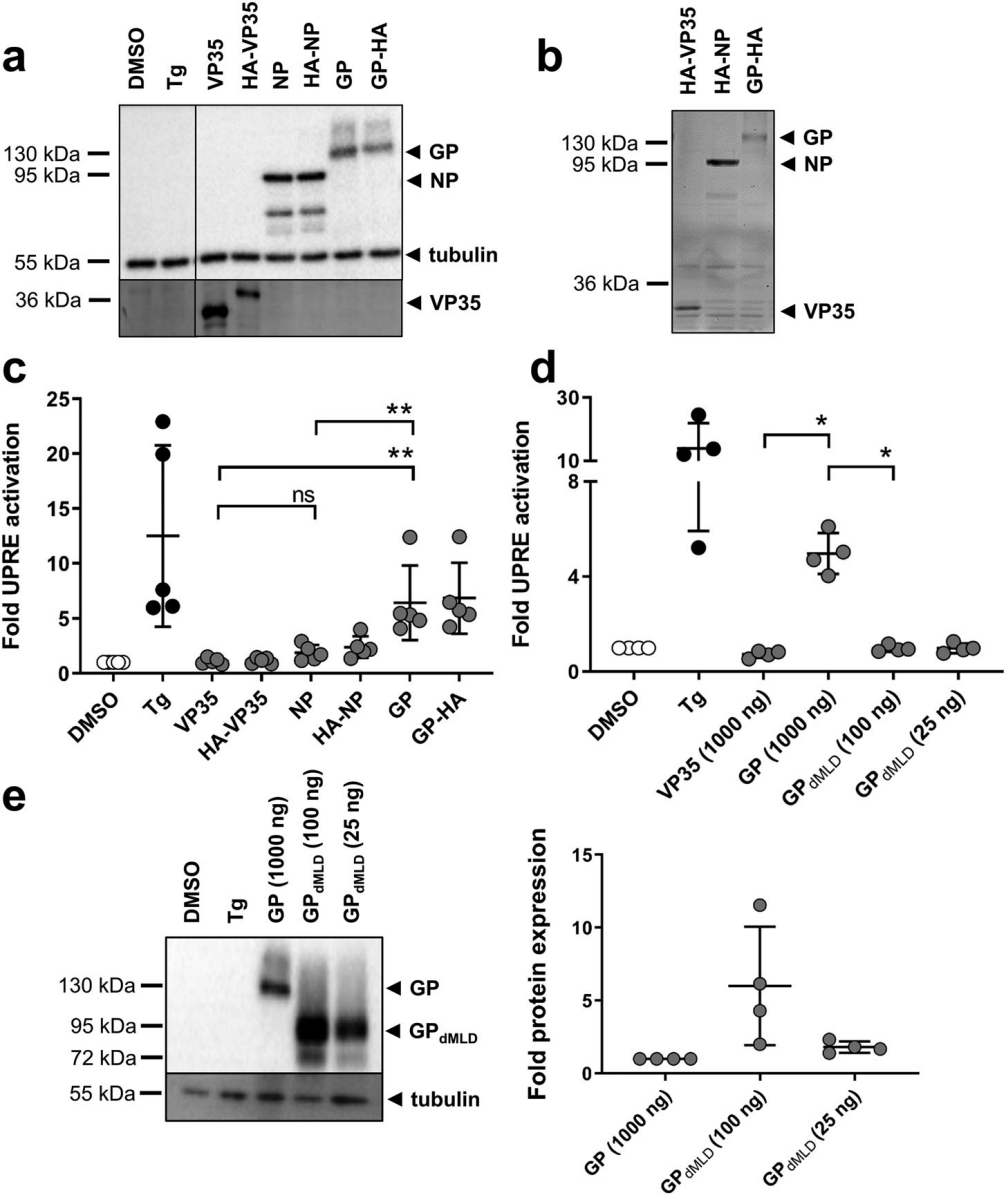
MARV GP activates the unfolded protein response element. (a) HuH7 cells were transfected with plasmids encoding firefly luciferase under the control of an UPRE promoter, with pGL4.73, which encodes Renilla luciferase, and with plasmids encoding NP, VP35 or GP. HuH7 cells transfected with the empty vector were treated with vehicle (DMSO) or with Tg. The cells were lysed at 48 h post transfection (p.t.), and equal amounts of the cell lysates were subjected to Western blotting using monoclonal antibodies against GP, NP and tubulin and polyclonal anti-VP35 serum. The experiment was performed five times; the results of one representative experiment are shown. (b) Equal amounts of cell lysates were subjected to SDS-PAGE, and the gels were subsequently incubated with anti-HA antibodies to detect HA-tagged viral proteins. (c) Cell lysates were analysed using luciferase assays. Firefly luciferase activity was normalized to Renilla activity, and the fold activation in comparison to the DMSO control (set to 1) was calculated. The experiment was performed five times. Statistical analysis was performed for wildtype proteins. (d) HuH7 cells were treated and transfected as described in (a) except that the amount of GP_dMLD_-expressing plasmid used for transfection was reduced (25 or 100 ng). The total amount of transfected plasmid was kept constant by the addition of empty vector. The experiment was performed four times. (e) Cell lysates were subjected to Western blotting using monoclonal antibodies to detect MARV GP and tubulin. Protein amount was quantified in each of the four independent experiments shown in d. Each circle represents a sample from an individual experiment, data are shown as the means ± SD.

HuH7 cells (2 × 10^5^ cells) and HAP cells (6 × 10^5^ cells) were seeded in 6-well plates and transfected with plasmids on the next day using TransIT-LT1 reagent (Mirus Bio LCC) according to the manufacturer's instructions. The following plasmids were transfected: 1 µg (6-well format) of p5xUPRE-GL3, which encodes firefly luciferase controlled by a UPRE promoter [8,30], and 0.1 µg of a plasmid encoding Renilla luciferase under the control of the SV40 early enhancer/promoter (pGL4.73, Promega) for normalization purposes. To stimulate UPRE-dependent reporter gene expression, cells were either infected with MARV (MOI = 1), treated with thapsigargin (Tg, Sigma-Aldrich, T9033) or treated with tunicamycin (Tu, Sigma-Aldrich, T7765). To analyse UPRE activation by MARV proteins, the cells were additionally transfected with pCAGGS-based plasmids encoding viral proteins. If combinations of two viral proteins were to be expressed, 0.5 µg (Figure 4(a)) or 1 µg (Figure 6(e)) of each plasmid was used. Single viral proteins were expressed by transfecting 1 µg of the appropriate plasmid (Figure 1 and Figure 4(c)). Transfection within the setting of an infectious virus-like particle assay (iVLP) was performed as described by Wenigenrath et al. [25]. The negative control samples were mock-infected and/or treated with vehicle (DMSO). Stimulation with Tg or Tu was performed 16 or 24 h before the cells were lysed. MARV infection of cells was performed at 24 h post-transfection (p.t.). The cells were lysed at 48 h p.t. or p.i. in passive lysis buffer (Promega). Luciferase assays were performed using the Beetle-Juice and Renilla-Juice BIG KITs (PJK). Renilla luciferase signals were used to normalize for transfection efficiency.

### XBP1 splicing RT–PCR

Cellular RNA was isolated using the RNeasy Mini Kit (QIAGEN) according to the manufacturer’s instructions (DNaseI digestion included). One µg of the eluted RNA was used for reverse transcription (RT) using the Omniscript® Reverse Transcription Kit (QIAGEN) and *XBP1*-specific (#3166: 5’-GTAAGCATCCAGTAGGCAGGAAG) forward primer for 1 h at 37°C. cDNA was purified (E.Z.N.A. ® DNA Probe Purification kit) and amplified using Taq Polymerase (Thermo Scientific) and primers designed to amplify 267 nucleotides including the splicing site of the *XBP1* mRNA (#3353: 5’-CATGGCCTTGTAGTTGAGAACCAGG; #3354: 5’-GGTCCAAGTTGTCCAGAATGC CCAA). Amplified *XBP1*-specific PCR fragments were purified and 10 µl were digested with *PstI* to distinguish variants of *XBP1* mRNA (*XBP1s* and *XBP1u*). Analyses of *XBP1*-mRNA species were performed by 4% agarose gel electrophoresis, staining with ethidium bromide and visualization by UV light.

### XBP1 splicing – protein

**Figure 2.**
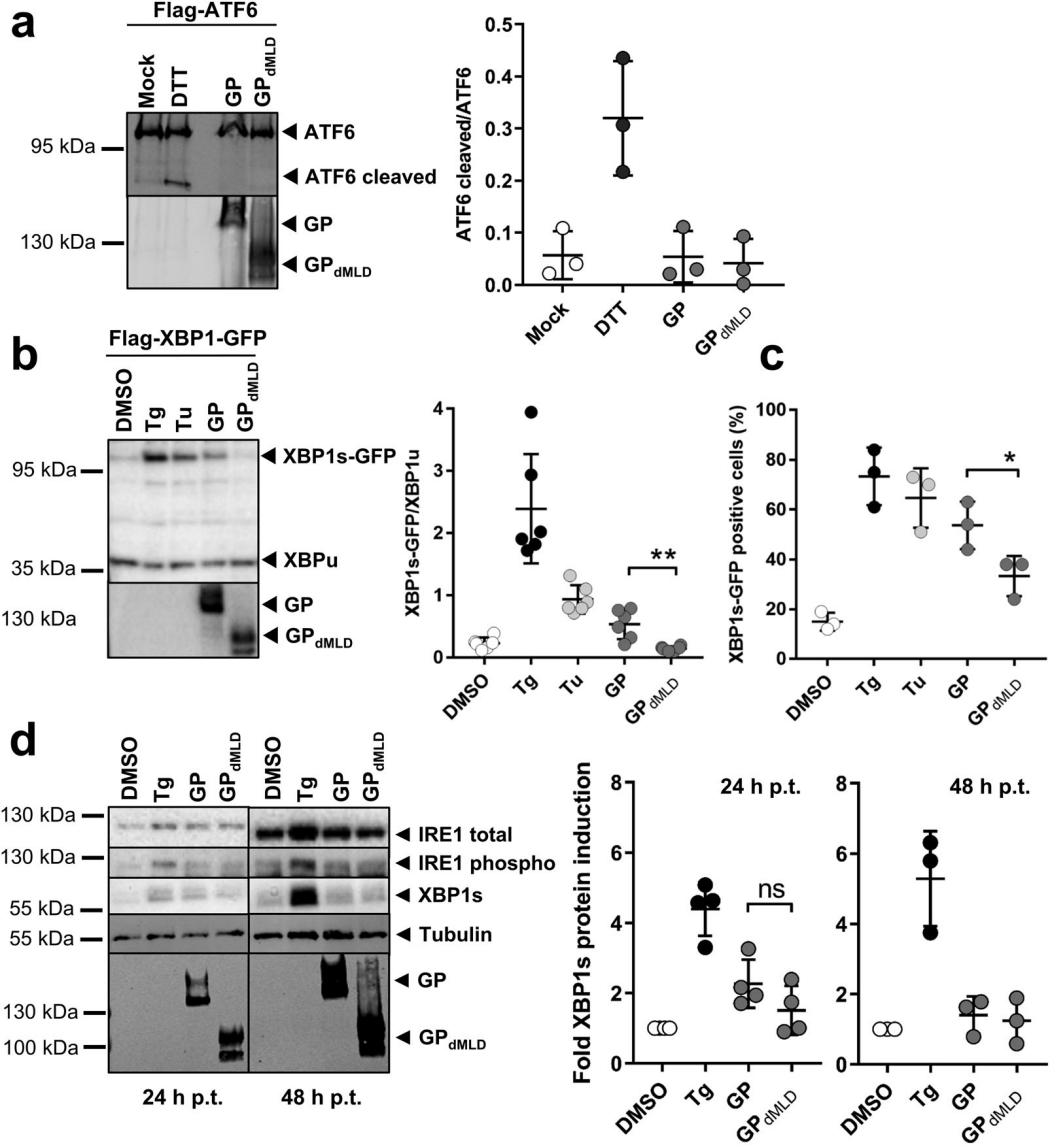
MARV GP activates UPR in an IRE1/XBP1-dependent manner. (a) HuH7 cells transfected with plasmids encoding Flag-ATF6 and GP or GP_dMLD_ (1 µg each) were lysed 48 h p.t. and analysed by Western blotting using an anti-Flag mouse monoclonal antibody and an Alexa680-conjugated anti-mouse antibody to detect full-length and cleaved (active) ATF6. MARV-specific goat serum and an IRdye800-conjugated anti-goat antibody were used to detect the viral proteins. Incubation of cells with 1 mM DTT for 30 min served as a positive control. Detection and quantification were performed using an Odyssey imaging system. The ratio of cleaved ATF6 protein to full-length ATF6 protein was calculated. The experiment was performed three times. (b) HuH7 cells were transfected with plasmids encoding Flag-XBP1-GFP, GP (1 µg), GP_dMLD_ (25 ng) or empty vector (DMSO, Tg, Tu). The total amount of transfected plasmid (2 µg in total) was kept constant by the addition of empty vector. XBP1 splicing was induced by 5 nM Tg or 300 nM Tu for 16 h. The cells were lysed at 48 h p.t. and analysed by Western blotting using monoclonal antibodies against the Flag-tag and GP and peroxidase-coupled secondary antibodies. XBP1s and XBP1u were quantified using the ChemiDoc imaging system, and the ratios of these proteins were calculated. The experiment was performed six times. (c) HuH7 cells that had been treated and transfected as explained in b were fixed 48 h p.t. and subjected to immunofluorescence analysis. DMSO, Tg and Tu: HuH7 cells were transfected with an mCherry-expressing plasmid instead of with empty vector and were treated as indicated in b. Viral proteins were stained using monoclonal protein-specific and fluorescently labelled secondary antibodies. XBP1s-GFP positive nuclei were counted in cells expressing the viral protein or mCherry in three independent experiments. The percentage of XBP1s-GFP positive nuclei is shown. Each circle represents the result from an individual experiment, data are shown as the means ± SD. (d) HuH7 cells were transfected with plasmids encoding GP (1 µg), GP_dMLD_ (200 ng) or mCherry (DMSO, Tg). The total amount of transfected plasmid (2 µg in total) was kept constant by the addition of mCherry plasmid. Cells were lysed at 24 and 48 h p.t. and subjected to Western blot analysis to detect endogenous IRE1 and XBP1s proteins using protein-specific antibodies detected by POD-coupled secondary antibodies. 24 and 48 h samples were analysed in parallel on the same blot afterwards tubulin and MARV GP were detected. XBP1s levels were quantified and presented as relative values to DMSO-treated cells (set to 1). The experiments were performed four (24 h) or three (48 h) times. Each circle represents a sample from an individual experiment, data are shown as the means ± SD.

HuH7 cells (2 × 10^5^ cells/6-well) were transfected with pCAGGS-Flag-XBP1-GFP [31] together with pCAGGS-mCherry (each 1 µg, total 2 µg) using the TransIT-LT1 reagent. The expression of XBP1s is dependent on the posttranscriptional splicing of *XBP1u* mRNA by IRE1. It excises 26 nucleotides from the *XBP1u* mRNA, resulting in a frame shift. To monitor this event, we fused GFP to XBP1s. If IRE1 is silent, Flag-XBP1u is expressed. When IRE1 is activated, *XBP1u* mRNA splicing leads to the expression of Flag-XBP1s-GFP. Both variants can be detected by the N-terminal Flag tag; XBP1s can be visualized by GFP. To stimulate XBP1 splicing, cells were treated with Tg (5 nM) or with Tu (300 nM) for 16 h or transfected with plasmids encoding MARV proteins. The cells were analysed by Western blotting and by fluorescence microscopy (transfected cells). Eight images obtained from each of three individual experiments were quantified by counting the number of cells positive for XBP1s and mCherry or a viral protein and the percentages of GFP-positive cells were calculated (Figure 2(C)).

### ATF6 cleavage assay

HuH7 cells were transfected as described above with the plasmid p3xFlag-ATF6 together with empty vector (pCAGGS) (2 µg plasmid DNA/6-well). To monitor ATF6 cleavage, we utilized the Flag-tagged ATF6 construct p3xFLAG-ATF6, a gift from Ron Prywes (Addgene plasmid #11975) [32]. To stimulate ATF6 cleavage, cells were treated with 1 mM dithiothreitol for 30 min (DTT, Sigma-Aldrich, D9779) or transfected with plasmids encoding MARV proteins. The cells were analysed by Western blotting at 48 h p.t.

### Western blot analysis

Whole-cell extracts were prepared using 1x SDS sample buffer [33] or if analysed for endogenous IRE1 and XBP1s using cell lysis buffer as described by Krähling et al. [33]. Cells were then treated with 10 µM MG132 1 or 2 h before the harvest. The proteins were separated on SDS-polyacrylamide gels and transferred to nitrocellulose membranes (Amersham Protran 0.45 NC). Blocking was performed in phosphate-buffered saline (PBS) containing 10% skim milk or as recommended by the manufacturer. Immunostaining was performed using primary antibodies diluted in PBS containing 1% (w/v) skim milk and 0.1% Tween-20: anti-NP 59-9-10 (1:4000), anti-GP 50-6-10 (1:100), anti-VP40 40-2-2 (1:4000), and anti-VP30 11-6-11 (1:1000), anti-MARV-VP35-2 (guinea pig, 1:500), anti-MARV goat serum (1:5000), anti-HA (rabbit, 1:500, Rockland, Cat. No. 600-401-384) and anti-Flag (rabbit, 1:500 (Sigma-Aldrich Cat. No. F7425) or mouse, 1:1000 (Sigma-Aldrich Cat. No. F3165)) were used to detect the proteins. A mouse monoclonal antibody (Clone DM 1A, 1:5000, Sigma-Aldrich) was used to detect α-tubulin. The following antibodies were used to detect endogenous IRE1 and XBP1s according to manufacturer’s instructions: #3294 and #83418 from Cell Signalling and #124945 from Abcam. Western blot detection and quantification were performed using POD-conjugated secondary antibodies (1:30,000), Image Lab™ software and the ChemiDoc™ XRS^+^ System (BIO-RAD) or with IRDye® 680 or IRDye® 800 secondary antibodies (1:5000) using the Odyssey® CLx imaging system.

### In-gel detection of proteins

Whole cell extracts were prepared and separated by SDS-PAGE as described above. Immunostaining was performed as described in the LI-COR® manual “In-Gel Western Detection Using Near-Infrared Fluorescence”. Anti-HA antibody (rabbit, 1:500, Rockland) was diluted in PBS containing 5% (w/v) BSA (Serva Electrophoresis GmbH, 11926) and 0.1% Tween-20 and applied overnight at 4°C. In-gel detection was performed using IRDye® 680 secondary antibody in PBS containing 5% (w/v) BSA and 0.1% Tween-20 (1:1000). Detection was performed using the Odyssey^®^ CLx imaging system.

### Immunofluorescence analysis

IFA was performed as described previously [29]. Viral proteins were detected using monoclonal antibodies against NP or GP (anti-NP 59-9-10, 1:100; anti GP 50-6-10, 1:20) in combination with an Alexa Fluor® 594-conjugated anti-mouse secondary antibody (1:500). DAPI (4’,6’-diamidino-2-phenylindole, 0.5 µg/ml) staining was used to visualize cell nuclei. Images were acquired on a Zeiss Axiophot upright fluorescence microscope (63x objective) using a Spot inside B/W QE digital camera (Visitron Systems, Puchheim, Germany) and VisiView image acquisition software.

### Co-immunoprecipitation analysis

HuH7 cells (2 × 10^5^ cells/6-well) were transfected with the plasmids pCAGGS-Flag-XBP1-GFP, pCAGGS-HA-VP30, pCAGGS-VP30-GFP [21], pCAGGS-IRE1 or with empty vector (pCAGGS) (a total of 2 µg plasmid DNA/well) using the TransIT-LT1 reagent. Co-IP analyses were performed as described previously [34] using anti-HA affinity gel agarose (Sigma-Aldrich, A2095). As changes to the published protocol, we performed precipitation for 3 h at 4°C, and each pellet was resuspended in 45 µl of 2x SDS sample buffer.

### Statistical analyses

All samples represent biological replicates. Sample sizes are evident in each figure. Each circle represents a sample from an individual experiment; the data are presented as the mean ± SD. Unpaired two-tailed *t*-tests were used to compare two sets of data for which Gaussian distribution could be assumed (Figure 2(c)). For most statistical analysis it could not be assumed that data are from a population that follows a Gaussian distribution; then the non-parametric statistical Mann–Whitney test was used to compare two sets of data with *n* ≥ 4 (Figure 1; Figure 2(b); Figure 4(a,c); Figure 7) or the Wilcoxon signed-rank test was used to compare one data set against a hypothetical value (Figure 5(b)) if sample size was *n* ≥ 6. The following significance levels were applied: *p* ≤ 0.05 = *; *p* ≤ 0.01 = **. All data were analysed using Prism version 8.1.1 (GraphPad software Inc., San Diego, CA).

## Results

### MARV GP activates the unfolded protein response element

Since MARV GP accumulates in the ER of infected cells, it was of interest to analyse whether expression of GP leads to activation of the UPR. We used a firefly luciferase-based reporter construct that contains a UPR *cis*-active promoter element regulated by the transcription factors XBP1s and ATF6 (p5xUPRE-GL3) [8]. Our system was calibrated using thapsigargin (Tg) or tunicamycin (Tu). Tg inhibits sarcoplasmic reticulum Ca^2+^ ATPases (SERCA) and thus halts the active transport of Ca^2+^ into the ER, leading to depletion of Ca^2+^ levels and UPR activation [35]. Tu blocks N-linked glycosylation, thereby interfering with protein folding, and causes UPR activation [36,37]. HuH7 cells were transfected with p5xUPRE-GL3 and a plasmid that expresses the Renilla luciferase under the control of the SV40 early enhancer/promoter (pGL4.73). The transfected cells were incubated with increasing concentrations of Tg or Tu. Treatment of the cells with either substance resulted in a concentration-dependent induction of UPRE (Figure S1). The lowest concentrations of Tg and Tu that reliably induced UPRE were 5 and 300 nM, respectively; these concentrations were used in further experiments except where otherwise indicated.

We next investigated whether ectopic expression of MARV GP influences the activity of UPRE. HuH7 cells were transfected with p5xUPRE-GL3 and pGL4.73 together with plasmids encoding MARV GP, VP35 or NP or HA-tagged versions of the viral proteins. Western blot analyses confirmed the expression of the viral proteins, and no obvious differences in the levels of expression of the wild-type and HA-tagged versions were observed (Figure 1(a)). Importantly, in-gel detection of HA-tagged proteins using an antibody against HA showed that the signal strengths of GP and VP35 were similar, whereas NP appeared to be expressed in higher amounts (Figure 1(b)). The luciferase activities of the same samples corresponding to the luciferase protein that accumulated over the past 48 h of expression were analysed; the results revealed that GP reliably activated the expression of the UPRE-controlled reporter gene in contrast to NP and VP35 (Figure 1(c)).

To determine whether the number of glycosylation acceptor sites of MARV GP influences UPRE activation, we employed a GP protein lacking the mucin-like domain (GP_dMLD_). Deletion of the MLD specifically removes most of the glycan acceptor sites [23,38]. Western blot analyses showed that after transfection of cells with the two plasmids at identical concentrations, the expression level of GP_dMLD_ was higher compared to that of GP although the activation of UPRE was reduced (Figure S2). Therefore, the amount of the GP_dMLD_ plasmid used in transfection was reduced to 100 or 25 ng (Figure 1(d,e)). Transfection of 25 ng resulted in the expression of similar amounts of GP_dMLD_ and GP proteins at 48 h after transfection (Figure 1(e)). Immunofluorescence analysis (IFA) of single cells expressing GP or GP_dMLD_ revealed that the two proteins showed comparable localization patterns and signal strengths (Figure S3). Under these conditions, GP_dMLD_ did not activate the UPRE reporter (Figure 1(d)). It is presumed that due to the reduced number of glycosylation sites in GP_dMLD_, the ER transit of GP_dMLD_ occurs more rapidly than that of GP; this is likely the reason why GP_dMLD_-expressing cells exhibit less ER stress. Taken together, the data show that intracellular expression of MARV GP leads to activation of the UPRE.

### MARV GP activates UPR in an IRE1/XBP1-dependent manner

It was of interest to determine whether GP-induced UPRE reporter activity was mediated by activation of ATF6 and/or XBP1s. The activation of these transcription factors was analysed by monitoring the ratio of XBP1s to XBP1u and the ratio of cleaved ATF6 to full-length ATF6 by Western blot analysis 48 h after transfection.

We treated HuH7 cells with dithiothreitol (DTT), which is known to induce ATF6 cleavage, as a positive control [32]. HuH7 cells were transfected with plasmids encoding Flag-ATF6 and GP or GP_dMLD_. The signals of full-length and cleaved ATF6 were quantified, and the ratio of the two proteins was calculated. While DTT treatment led to an increase in the proportion of cleaved ATF6 compared to mock-treated cells, the expression of MARV proteins did not (Figure 2(a)).

Activation of XBP1 was monitored by quantifying the levels of the XBP1s and XBP1u proteins and calculating their ratio. Cells were transfected with a plasmid encoding XBP1u or XBP1s-GFP in dependence of the IRE1 activity in the cells. XBP1s-GFP is expressed only upon splicing of *XBP1u* mRNA (Figure S4a). Treatment of HuH7 cells with Tg or Tu, known inducers of XBP1s, increased the XBP1s-GFP/XBP1u ratio compared to the DMSO control (Figure 2(b)). Expression of GP also led to an increase in this ratio, whereas GP_dMLD_ did not. To further support this result, we analysed the effect of the expression of GP and GP_dMLD_ on the number of cells expressing XBP1s-GFP in the nucleus. In the presence of GP, the number of XBP1s-GFP expressing cells was significantly increased when compared to GP_dMLD_ (Figure 2(c) and S4b).

To analyse expression levels of endogenous proteins of the IRE1-XBP1 axis, IRE1 (total and phosphorylated) and XBP1s were monitored in cells expressing GP and GP_dMLD_ at 24 and 48 h p.t. Two hours before the cells were lysed they were treated with the proteasomal inhibitor MG132 in order to prevent degradation of IRE1 and XBP1s. Western blot analysis showed that Tg-treatment increased IRE1 phosphorylation and XBP1s expression (Figure 2(d)), whereas ectopic expression of viral proteins had no significant effect.

Taken together, the data show that intracellular expression of MARV GP but not GP_dMLD_ leads to activation of the UPRE by IRE1 via XBP1s-dependent signalling. This could be monitored by UPRE activation and analysing ectopically expressed ATF6 and XBP1u/s.

### IRE1-dependent UPRE is not activated during MARV infection

**Figure 3.**
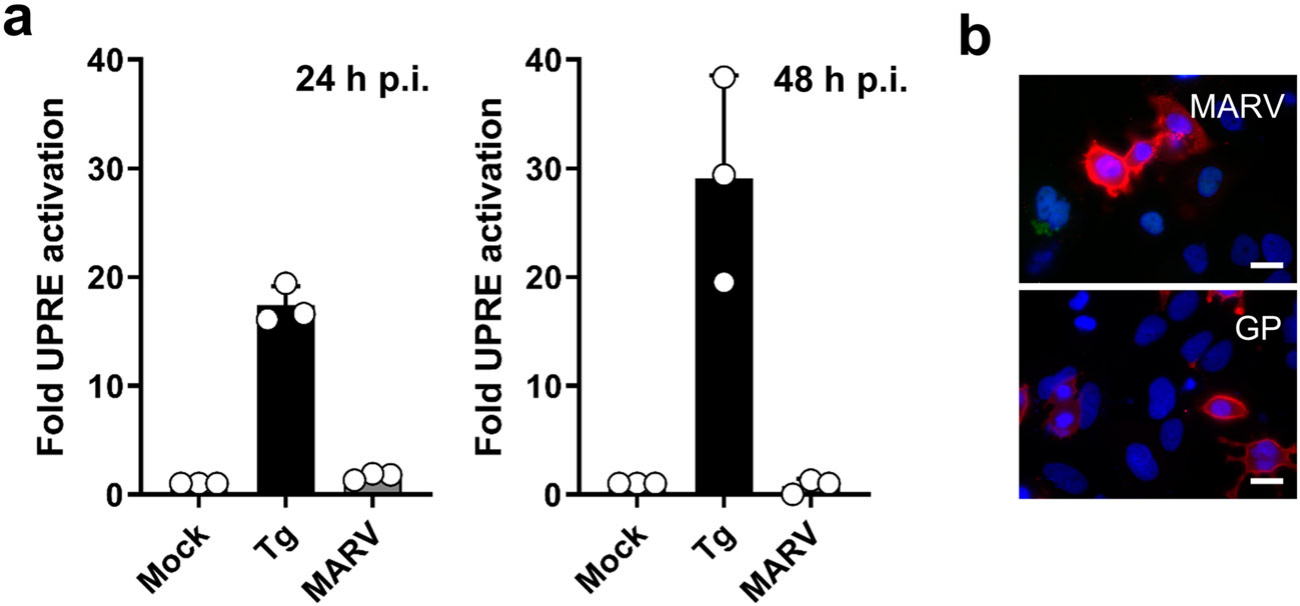
MARV infection does not induce UPRE. (a) The UPRE firefly luciferase assay was performed as described in the legend to Figure 1. At 24 h p.t. the cells were infected with MARV at a MOI of 1. Tg (300 nM for 24 h) was used to activate the UPRE reporter. The cells were lysed at 24 or 48 h post infection (p.i.) and analysed using the luciferase assay. The experiments were performed three times. Each circle represents a sample from an individual experiment, data are shown as the means ± SD. (b) HuH7 cells were infected with MARV (see above) or transfected with plasmids encoding GP as described in the legend to Figure 1. The cells were fixed after 48 h and subjected to IFA using a monoclonal antibody against GP. The photomicrographs were obtained using the same exposure times. DAPI staining labels cell nuclei. Scale bar = 25 µm.

To analyse whether UPRE was also activated during MARV infection, HuH7 cells were transfected with p5xUPRE-GL3 and pGL4.73 (see Figure 1). One day after transfection, the cells were infected with MARV at a MOI of one. Luciferase assays at 24 and 48 h p.i. showed that MARV infection did not induce reporter gene activity controlled by the UPRE, indicating that neither XBP1s nor ATF6 were activated (Figure 3(a)). The expression levels of GP in transfected and infected cells at 48 h p.i. were compared by IFA (Figure 3(b)). These analyses revealed that the expression levels of GP were similar in infected and in transfected cells. These results suggested that the GP-dependent UPRE activation was counter-regulated during MARV infection.

### VP30 reduces GP- and Tg-induced UPRE-dependent signalling

**Figure 4.**
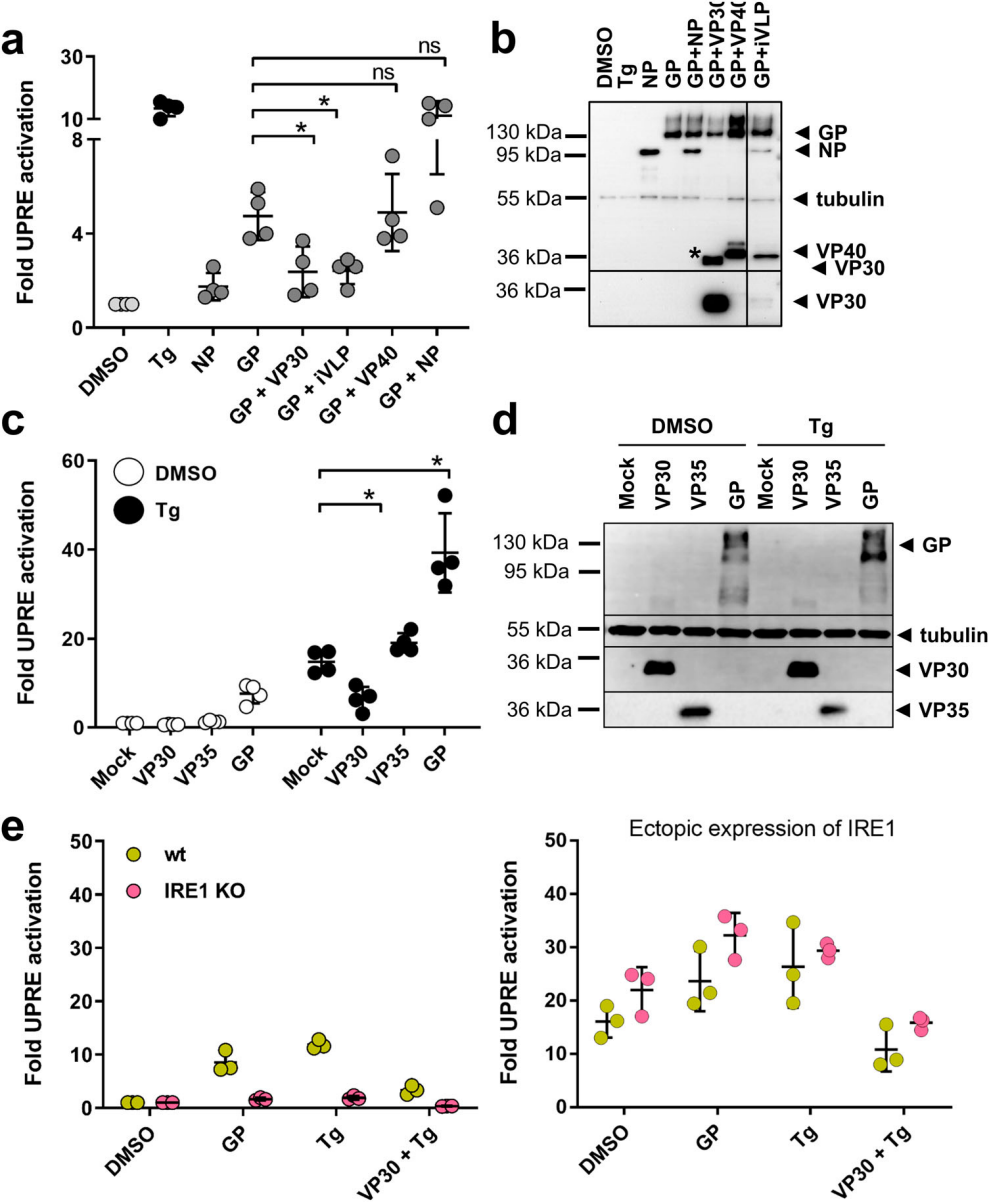
VP30 reduces GP- and Tg-induced UPRE-dependent signalling. (a) HuH7 cells were transfected with plasmids encoding the indicated MARV proteins and the UPRE-specific luciferase reporter plasmids as described in the legend to Figure 1. To express viral proteins, 0.5 µg of each plasmid was used in the transfection. In the iVLP setting, which involved the use of a combination of plasmids encoding all MARV proteins, the plasmid amounts used in transfection were as described by Wenigenrath et al. [25]. The experiment was repeated 4 times. (b) Equal amounts of lysates of transfected HuH7 cells were subjected to Western blot analysis using monoclonal antibodies. NP, GP, VP30, and tubulin were detected simultaneously; VP40 was stained afterwards on the same blot. The asterisk indicates remaining VP30 staining; irrelevant lines have been removed. (c) VP30-dependent reduction of Tg-induced UPR. Tg (5 nM) was used to induce UPRE-dependent reporter gene expression in VP30-, VP35-, and GP-expressing cells that had been transfected as described in the legend to Figure 1. The experiment was repeated 4 times. (d) Western blot analysis of cell lysates obtained from c. VP35 was stained with a polyclonal antibody against VP35; GP, VP30, and tubulin were detected afterwards on the same blot using monoclonal antibodies. Each circle represents a sample from an individual experiment, data are shown as the means ± SD. (e) To analyse UPRE-dependent luciferase activity, HAP1 cells (wt, shown in yellow) or HAP1 IRE1 KO cells (shown in pink) were transfected, treated and harvested as described for HuH7 cells. To restore IRE1 signalling in KO cells, the KO cells were transfected with a plasmid encoding IRE1 (100 ng); The cells were treated either with vehicle (DMSO) or with 5 nM Tg for 16 h. The experiments were performed three times. Each circle represents a sample from an individual experiment, data are shown as the means ± SD.

To determine whether MARV proteins counteract GP-induced UPRE activity, p5xUPRE-GL3, pGL4.73 and the GP-encoding plasmid were transfected along with plasmids coding for NP, VP30 or VP40. Co-expression of VP30 significantly reduced the UPRE activation triggered by GP (Figure 4(a)). Surprisingly, co-expression of GP with NP led to an increase in reporter activity. In addition, all MARV proteins were ectopically expressed, which is equivalent to the conditions used for the production of infectious virus-like particles (iVLPs) [25]. Under these conditions, UPRE activity was also significantly reduced, supporting the idea that VP30 also exerts a UPRE-balancing activity in MARV-infected cells (Figure 4(a)). Moreover, although VP30 levels are much lower in the iVLP setting than during co-expression of VP30 alone, reduction in GP-induced UPRE is almost equivalent suggesting that other viral factors contribute to the UPRE balancing effect. Western blot analyses show the expression of the viral proteins (Figure 4(b)).

To further characterize the UPRE-inhibitory effect of VP30, we tested whether VP30 was also able to dampen activation by Tg. VP30-expressing cells transfected with the UPRE-luciferase reporter p5xUPRE-GL3 were treated with Tg for 16 h, and their luciferase activity was determined. In the presence of VP30, Tg-induced UPRE activity was significantly reduced (Figure 4(c)). In contrast, UPRE stimulation by Tg was enhanced by the expression of GP. Expression of the viral proteins was verified by Western blot analysis (Figure 4(d)).

To support the finding that MARV GP induces the IRE1-dependent pathway we utilized IRE1 knock-out (KO) cell lines. Treatment of the parental HAP1 (wt) and IRE1 KO cells with increasing concentrations of Tg and Tu resulted in the concentration-dependent activation of the UPRE in the parental cells but not in the IRE1 KO cells, showing that the lack of IRE1 prevents the activation of UPRE by Tg and Tu (Figure S5). This result suggests that UPRE activation in HAP1 cells is mainly mediated by IRE1. As in HuH7 cells, 5 nM Tg reliably induced UPRE reporter activity in HAP1 cells, so that in the following experiments 5 nM Tg were used.

The ability of GP to activate UPRE in IRE1 KO cell lines was analysed using the UPRE luciferase reporter assay. The results of this experiment showed that Tg treatment and ectopic expression of GP induced UPRE activity in HAP1 cells but not in IRE1 KO cells (Figure 4(e)). When the KO cell line was provided with ectopically expressed IRE1, its ability to respond to GP and Tg was restored (Figure 4(e)). These results substantiate our findings in HuH7 cells and confirm the GP-driven IRE1-dependent activation of the UPRE and the balancing effect of VP30 on the UPRE activated by Tg treatment.

Taken together, the results show that MARV has evolved a balancing function to reduce the GP-dependent activation of UPRE. MARV VP30 not only reduces UPRE signalling induced by GP but also down-regulates UPRE activation by Tg.

### VP30 co-precipitates XBP1u protein only in the presence of RNA

**Figure 5.**
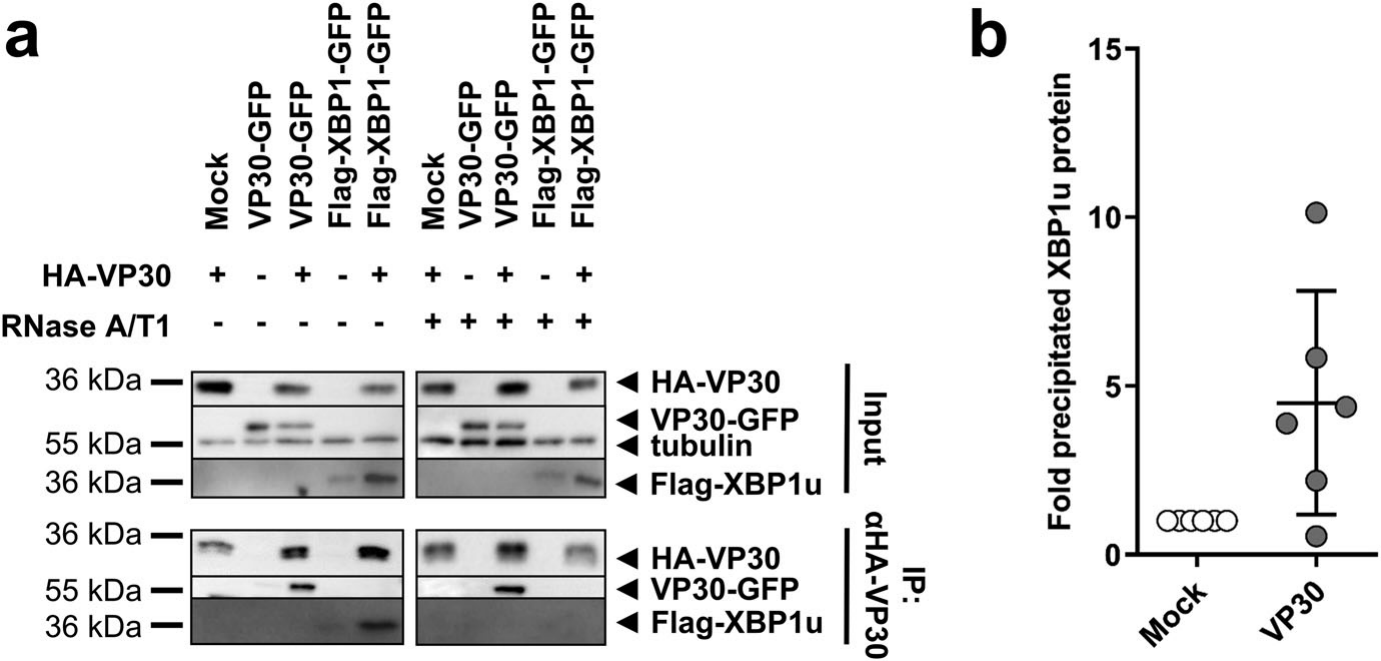
VP30 co-precipitates XBP1u protein in the presence of RNA. (a) HuH7 cells were transfected with plasmids expressing Flag-XBP1, VP30-GFP and HA-VP30. The cells were lysed 48 h p.t. and expression of ectopically expressed proteins was checked (input). The remaining lysate was subjected to co-immunoprecipitation analysis using anti-HA agarose according to Biedenkopf et al. [34]. (b) The amount of XBP1u precipitated in the presence of VP30 was compared with the amount precipitated in the absence of VP30 (set to 1). The amount of precipitated XBP1u protein was normalized to the expressed XBP1u (input) according to the tubulin content of the lysate. The experiment was performed six times. Each circle represents a sample from an individual experiment, data are shown as the means ± SD.

To determine the mechanism by which VP30 balances UPRE activity, we examined whether VP30 interacts directly with IRE1 or XBP1u or XBP1s. For this purpose, HuH7 cells were transfected with plasmids encoding HA-VP30, VP30-GFP and Flag-XBP1 or IRE1, and co-immunoprecipitation (co-IP) of cell lysates at 48 h p.t. was performed. Western blot analysis confirmed the expression of all ectopically expressed proteins (Figure 5(a), S6a and b, input). Using anti-HA agarose to precipitate HA-tagged VP30, it was possible to co-precipitate XBP1u protein (Figure 5(a), lane 5) but not XBP1s (Figure S6a) or IRE1 (Figure S6b). VP30-GFP could also be co-precipitated with HA-tagged VP30, which was expected since MARV VP30, like EBOV VP30, is presumed to undergo homooligomerization (Figure 5(a), lane 3). Quantification of six independent experiments revealed that the efficiency of co-precipitation of XBP1u by HA-VP30 was highly variable (Figure 5(b)). To determine whether the VP30-XBP1u interaction was dependent on the presence of RNA, RNase A/T1 was added to the co-precipitation reaction. Although in the presence of RNase A/T1 VP30-GFP was still co-precipitated with HA-VP30, the interaction of VP30 with XBP1u was no longer detected under these conditions, indicating that the VP30-XBP1u interaction depends on the presence of RNA. It has been reported recently that the self-interaction of Ebola virus VP30 is independent of the presence of RNA [34]; this is obviously also true for MARV VP30.

### MARV propagation is affected by IRE1 signalling

**Figure 6.**
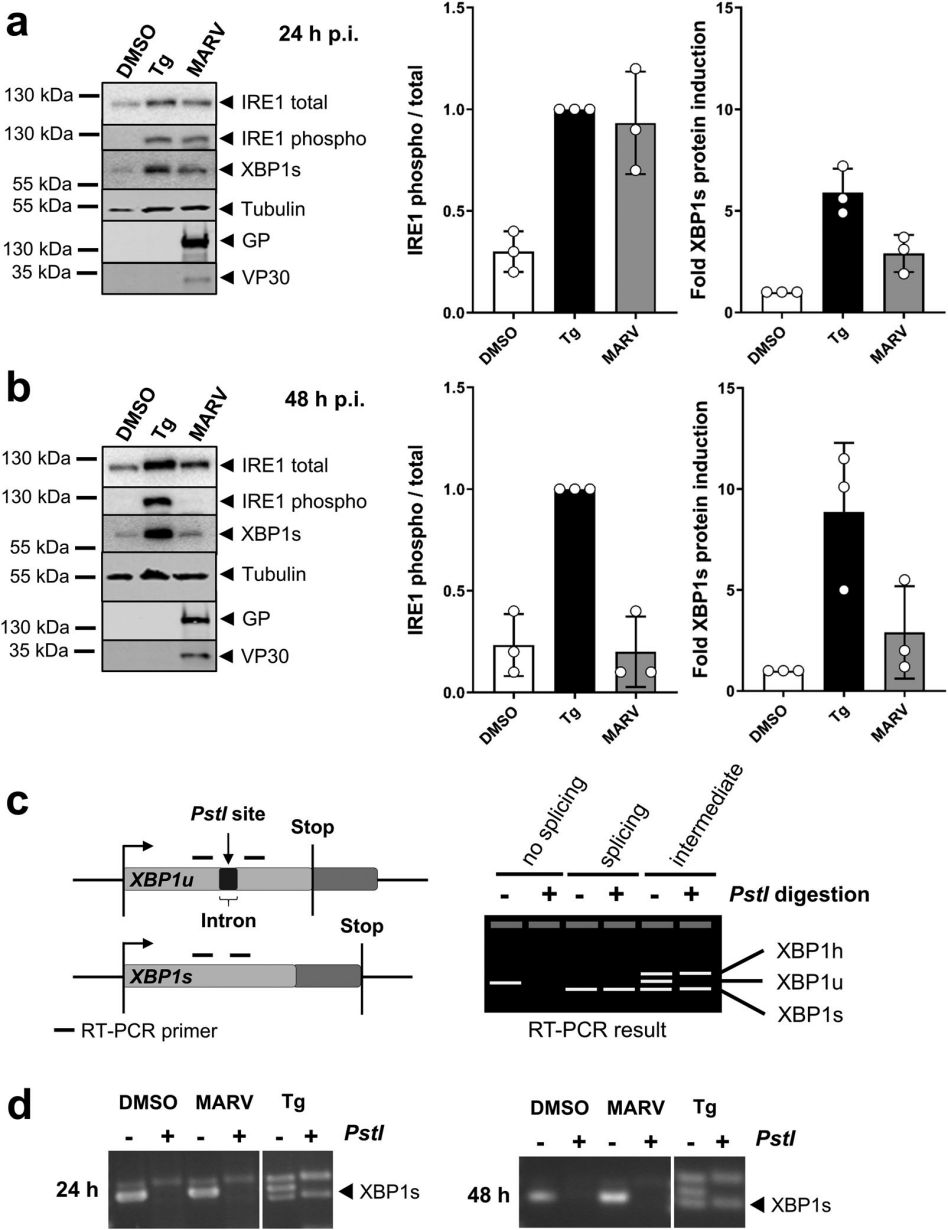
IRE1-dependent signalling during MARV infection. (a, b) HuH7 cells were infected with MARV at a MOI of 1. Cells were lysed at 24 h (a) and 48 h p.i. (b) and subjected to Western blot analysis to detect endogenous IRE1 (total and phosphorylated) and XBP1s proteins as explained in the legend to Figure 3. Total and phosphorylated IRE1 was quantified in each sample, compared to each other and set in relation to Tg-treated samples (set to 1). XBP1s levels were quantified and presented as relative values to DMSO-treated cells (set to 1). The experiments were performed three times. Each circle represents a sample from an individual experiment, data are shown as the means ± SD. (c) Scheme of *XBP1*-specific mRNAs and RT-PCR results. If there is no IRE1 activity, *XBP1u* mRNA is not spliced by IRE1; the *PstI* restriction site is available and the PCR product can be digested. Under conditions of IRE1 activation, *XBP1u* is spliced; *PstI* restriction site is lost and the PCR product cannot be digested by the enzyme. Intermediate phenotype: *XBP1u* is partially spliced; As published by others [39] we detect that *XBP1u* and *XBP1s* form a hybrid (XBP1 h, confirmed by sequencing) that is visible in the agarose gel and resistant to digestion. (d) XBP1-specific RT-PCR of RNA derived from HuH7 cells infected with MARV at a MOI of 1 for the indicated times. XBP1 splicing was induced using 5 nM Tg.

We then monitored expression levels of endogenous IRE1, phosphorylated IRE1 and XBP1s in MARV-infected cells at 24 and 48 h p.i. Cells were treated with the proteasomal inhibitor MG132 at one hour before lysis in order to prevent degradation of IRE1 and XBP1s. Western blot analysis showed that IRE1 is phosphorylated in MARV-infected cells at 24 h (Figure 6(a)) but not at 48 h p.i. (Figure 6(b)). XBP1s levels increased slightly at 24 and 48 h p.i. compared to levels monitored in DMSO-treated cells. These results correlated with the levels of GP which were higher at 24 h than at 48 h p.i. (Figure 6 and S7a). Analysis of infection revealed that 100% of the cells were infected at 24 h p.i. (Figure S7b). Interestingly, RT–PCR analyses of *XBP1* mRNAs (Figure 6(c)) showed no increase of *XBP1u* splicing upon MARV infection (Figure 6(d)). In contrast, cells stimulated with Tg, clearly displayed induction of *XBP1u* splicing, indicated by the presence of *XBP1s* mRNA (Figure 6(d)). Taken together, these data support a model that peak protein production of most likely GP at the ER during the first replication cycle of MARV infection, induces IRE1 signalling, as monitored by IRE1 phosphorylation at 24 h p.i. In contrast, XBP1 splicing was not detected and XBP1s protein levels were only moderately increased.

**Figure 7.**
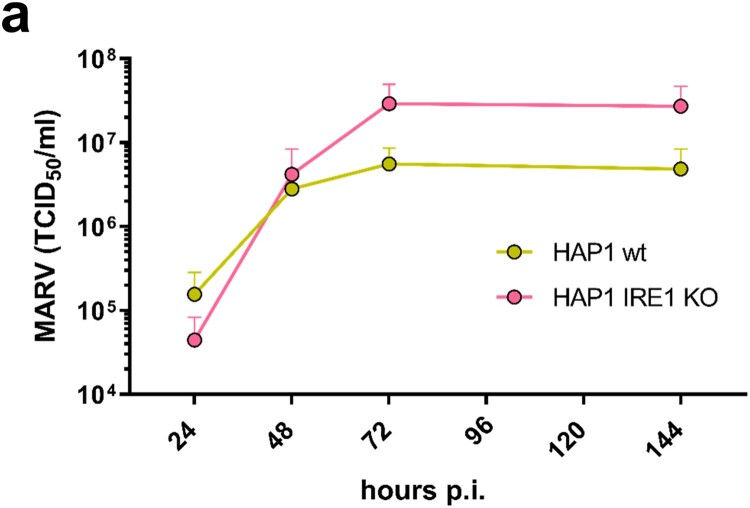
MARV propagation is affected by IRE1. (a) HAP1 cells (wt, shown in yellow) and HAP1 IRE1 KO cells (shown in pink) (6 × 10^5^ cells) were infected with MARV at a MOI of 0.1; the cell culture supernatants were collected after 24, 48, 72 and 144 h p.i. and analysed for the presence of infectious MARV by TCID_50_ assays.

To further characterize the role of IRE1 during MARV infection, we analysed the progression of infection in HAP1 parental (wt) and IRE1 KO cells over a period of 6 days. As shown in Figure 7, MARV titres were higher in KO cells at days three and six of infection leading to the assumption that IRE1 deficiency has a positive effect on MARV propagation in cell culture over time.

## Discussion

Acute viral infections have dramatic effects on the cellular pathways that are hijacked to support virus growth. Interestingly, although cellular metabolism is often massively affected, cellular defense mechanisms are out-maneuvered by viral effector functions that have been developed during virus adaptation to the host [1]. The best-studied example of this is the cellular interferon system [2]; numerous examples show that efficient virus infection requires sophisticated manipulation at the level of interferon production, signalling and the expression of interferon-stimulated genes. Likewise, viruses also address the UPR to make use of its beneficial functions, for example by upregulating the expression of chaperones and by counteracting potentially antiviral functions such as terminal UPR, which leads to apoptosis [40].

A previous study showed that MARV GP accumulates in the ER, from which it is only slowly released for further transport to the plasma membrane [29]. It was, therefore, no surprise that expression of GP activated UPRE in the transfected cells. Removal of the mucin-like domain of GP, which contains most of the protein’s O- and N-glycosylation sites [23], nearly abolished UPRE activation by GP (Figures 1 and 2). Our results suggest that attachment and processing of the multiple glycosylation sites requires GP to be retained in the ER for a long period of time and that this contributes significantly to the UPRE-inducing activity of GP.

The expression of several viral glycoproteins results in UPR activation. For example, accumulation of the viral surface glycoproteins F and H in the ER of cells infected with canine distemper virus was shown to activate ER stress [41]. Additionally, the accumulation of the spike protein of severe acute respiratory syndrome coronavirus (SARS-CoV) leads to activation of the UPR [42]. In both cases, the UPR was activated both by infection and by ectopic expression of the respective glycoproteins. In contrast, the ectopic expression of MARV GP, but not MARV infection itself, resulted in UPRE activation, although similar amounts of GP were present in infected and transfected cells (Figure 3(b)). Explaining this unexpected result, we were able to demonstrate that the MARV transcription factor VP30 weakened the GP-dependent activation of UPRE. Additionally, UPRE activation by Tg was down-regulated by VP30.

VP30-mediated inhibition of the GP-induced effect could not be shown at the level of splicing of endogenous XBP1. This was due to the difficulty to clearly detect the GP-induced increase in endogenous XBP1s (Figure 2(d)). A possible explanation for this seemingly contradictory result is that the GP-mediated UPRE activation was monitored by measuring the accumulated activity of synthesized luciferase at 48 h p.t. However, in order to detect the rapidly degraded XBP1s, cells were treated with the proteasomal inhibitor MG132 for 2 h at the end of the incubation period [39,43]. Therefore, this assay mainly monitored the status of the last 2 h of the experiment instead of the whole period of 48 h.

VP30, a putative zinc-binding protein, enhances MARV transcription [25,44] and is essential for the rescue of recombinant MARV [45]. Relevant to this, siRNA studies have shown that down-regulation of VP30 leads to reduced expression of other filoviral proteins in infected cells, highlighting an important regulatory function of VP30 [46]. To our knowledge, VP30 does not interact directly with GP such an interaction might have explained its inhibitory effect. It was therefore assumed that VP30 directly influences IRE1 or downstream molecules in this signalling pathway. Consistent with this, we observed the RNA-dependent interaction of XBP1u and VP30 (Figure 5(a,b)) but not with XBP1s or IRE1 (Figure S6). Future analyses are needed to investigate the mechanistic details of the binding. Cytoplasmic splicing of the *XBP1u* mRNA by IRE1 at the ER membrane results in the translation of XBP1s, which is then transported into the nucleus, where it activates UPRE-controlled genes. Several studies have described a translational pausing mechanism that ensures efficient splicing of *XBP1u* by IRE1. During translational pausing, the nascent XBP1u protein/mRNA/ribosome complex is recruited to the ER membrane, where IRE1-mediated splicing of the *XBP1u* mRNA is initiated [10,47].

While it is possible that VP30 affects the localization, stability, or abundance of XBP1u, leading to an inhibition of IRE1-dependent UPRE activation, we now hypothesize that VP30 might interact with *XBP1u* mRNA and thus with the entire XBP1u/*XBP1u* mRNA/ribosome complex. The exact mechanism of such an effect must be investigated in further experiments.

Although many viruses activate UPR, the way in which each virus addresses this cellular response is different. Some viruses seem to benefit from UPR, whereas the growth of others is impaired [13,48]. Other viruses do not activate UPR because they have developed strategies to suppress UPR. For example, herpes simplex virus-1 (HSV-1) suppresses IRE1-dependent UPR at early stages of infection through the action of the viral protein UL41, which degrades *XBP1* mRNA via its RNase activity [49]. Su et al. showed that the kinase activity of IRE1 was beneficial to HSV-1 infection, whereas the RNase activity of IRE1 was detrimental [50]. Furthermore, the kinase activity of IRE1 results in the activation of c-Jun N-terminal kinase, which enhances the replication of HSV-1. Human and murine cytomegaloviruses (HCMV and MCMV) activate and manipulate the UPR to promote its pro-survival activity. UPR is inhibited by the M50 protein (MCMV) and the UL50 protein (HCMV), which target IRE1 for degradation [51]. Interestingly, deletion of XBP1u/s impedes MCMV gene expression, thereby causing a kinetic delay in infection [14]. These examples indicate that herpesviruses specifically regulate UPR at certain points in their replication cycles and suggest that tight regulation of the UPR is essential for efficient viral infection. We hypothesize that MARV also time-dependently regulates the IRE1 signalling for its own purpose. During the first 24 h p.i., which corresponds to the first MARV replication cycle, it may be that viral replication is more efficient when IRE1 is present and active (Figure 6(a), Figure 7). At this time point after infection, IRE1 is phosphorylated (Figure 6(a)), but XBP1s expression is only slightly increased. We believe that VP30 weakens the activation of the cascade by a so far unknown mechanism to achieve optimal conditions for viral propagation during its first replication cycle. Later in the course of infection the IRE1 KO seems to support viral infection. Further analyses are required to characterize the contribution of IRE1 and its different functions [52] to MARV replication in more detail.

In summary, we have shown that MARV infection does not induce UPRE-dependent reporter gene expression but seems to tightly regulate IRE1 phosphorylation and XBP1s expression. The ectopic expression of MARV GP results in UPRE activation. These at a first glance contradictory result are remedied by the discovery that MARV VP30 has an inhibitory effect on UPRE activity, thus counteracting GP-dependent activation. In conclusion, MARV seems to make use of and balances the IRE1-dependent signalling to create optimal conditions for its multiplication.

## Supplementary Material

Supplemental Material

## References

[1] Iranpour M, Moghadam AR, Yazdi M, et al. Apoptosis, autophagy and unfolded protein response pathways in arbovirus replication and pathogenesis. Expert Rev Mol Med. 2016;18:e1. DOI:10.1017/erm.2015.19. 26781343 PMC4836210

[2] Schulz KS, Mossman KL. Viral evasion strategies in type I IFN signaling – a summary of recent developments. Front Immunol. 2016;7:498. DOI:10.3389/fimmu.2016.00498. 27891131 PMC5104748

[3] Ron D, Walter P. Signal integration in the endoplasmic reticulum unfolded protein response. Nat Rev Mol Cell Biol. 2007;8:519–529. DOI:10.1038/nrm2199. 17565364

[4] Shore GC, Papa FR, Oakes SA. Signaling cell death from the endoplasmic reticulum stress response. Curr Opin Cell Biol. 2011;23:143–149. DOI:10.1016/j.ceb.2010.11.003. 21146390 PMC3078187

[5] Sano R, Reed JC. ER stress-induced cell death mechanisms. Biochim Biophys Acta. 2013;1833:3460–3470. DOI:10.1016/j.bbamcr.2013.06.028. 23850759 PMC3834229

[6] Korennykh A, Walter P. Structural basis of the unfolded protein response. Annu Rev Cell Dev Biol. 2012;28:251–277. DOI:10.1146/annurev-cellbio-101011-155826. 23057742

[7] Takayanagi S, Fukuda R, Takeuchi Y, et al. Gene regulatory network of unfolded protein response genes in endoplasmic reticulum stress. Cell Stress Chaperones. 2013;18:11–23. DOI:10.1007/s12192-012-0351-5. 22802018 PMC3508129

[8] Wang Y, Shen J, Arenzana N, et al. Activation of ATF6 and an ATF6 DNA binding site by the endoplasmic reticulum stress response. J Biol Chem. 2000;275:27013–27020. DOI:10.1074/jbc.M003322200. 10856300

[9] Yamamoto K, Yoshida H, Kokame K, et al. Differential contributions of ATF6 and XBP1 to the activation of endoplasmic reticulum stress-responsive cis-acting elements ERSE, UPRE and ERSE-II. J Biochem. 2004;136:343–350. DOI:10.1093/jb/mvh122. 15598891

[10] Yanagitani K, Kimata Y, Kadokura H, et al. Translational pausing ensures membrane targeting and cytoplasmic splicing of XBP1u mRNA. Science. 2011;331:586–589. DOI:10.1126/science.1197142. 21233347

[11] Ambrose RL, Mackenzie JM. West Nile virus differentially modulates the unfolded protein response to facilitate replication and immune evasion. J Virol. 2011;85:2723–2732. DOI:10.1128/JVI.02050-10. 21191014 PMC3067947

[12] Fischl W, Bartenschlager R. Exploitation of cellular pathways by dengue virus. Curr Opin Microbiol. 2011;14:470–475. DOI:10.1016/j.mib.2011.07.012. 21798792

[13] Hassan IH, Zhang MS, Powers LS, et al. Influenza A viral replication is blocked by inhibition of the inositol-requiring enzyme 1 (IRE1) stress pathway. J Biol Chem. 2012;287:4679–4689. DOI:10.1074/jbc.M111.284695. 22194594 PMC3281634

[14] Drori A, Messerle M, Brune W, et al. Lack of XBP-1 impedes murine cytomegalovirus gene expression. PLoS One. 2014;9:e110942, DOI:10.1371/journal.pone.0110942. 25333725 PMC4205010

[15] Ligon BL. Outbreak of Marburg hemorrhagic fever in Angola: a review of the history of the disease and its biological aspects. Semin Pediatr Infect Dis. 2005;16:219–224. doi: 10.1053/j.spid.2005.05.001 16044395 PMC7130051

[16] Kolesnikova L, Muhlberger E, Ryabchikova E, et al. Ultrastructural organization of recombinant Marburg virus nucleoprotein: comparison with Marburg virus inclusions. J Virol. 2000;74:3899–3904. doi: 10.1128/JVI.74.8.3899-3904.2000 10729166 PMC111900

[17] Hoenen T, Shabman RS, Groseth A, et al. Inclusion bodies are a site of Ebola virus replication. J Virol. 2012;86:11779–11788. DOI:10.1128/JVI.01525-12. 22915810 PMC3486333

[18] Becker S, Rinne C, Hofsäss U, et al. Interactions of Marburg virus nucleocapsid proteins. Virology. 1998;249:406–417. DOI:10.1006/viro.1998.9328. 9791031

[19] Bharat TA, Riches JD, Kolesnikova L, et al. Cryo-electron tomography of Marburg virus particles and their morphogenesis within infected cells. PLoS Biol. 2011;9:e1001196, DOI:10.1371/journal.pbio.1001196. 22110401 PMC3217011

[20] Kolesnikova L, Ryabchikova E, Shestopalov A, et al. Basolateral budding of Marburg virus: VP40 retargets viral glycoprotein GP to the basolateral surface. J Infect Dis. 2007;196(Suppl 2):S232–S236. DOI:10.1086/520584. 17940954

[21] Schudt G, Kolesnikova L, Dolnik O, et al. Live-cell imaging of Marburg virus-infected cells uncovers actin-dependent transport of nucleocapsids over long distances. Proc Natl Acad Sci USA. 2013;110:14402–14407. DOI:10.1073/pnas.1307681110. 23940347 PMC3761630

[22] Becker S, Klenk HD, Muhlberger E. Intracellular transport and processing of the Marburg virus surface protein in vertebrate and insect cells. Virology. 1996;225:145–155. DOI:10.1006/viro.1996.0582. 8918541

[23] Geyer H, Will C, Feldmann H, et al. Carbohydrate structure of Marburg virus glycoprotein. Glycobiology. 1992;2:299–312. doi: 10.1093/glycob/2.4.299 1421752 PMC7108560

[24] Krähling V, Dolnik O, Kolesnikova L, et al. Establishment of fruit bat cells (Rousettus aegyptiacus) as a model system for the investigation of filoviral infection. PLoS Negl Trop Dis. 2010;4:e802. DOI:10.1371/journal.pntd.0000802. 20808767 PMC2927428

[25] Wenigenrath J, Kolesnikova L, Hoenen T, et al. Establishment and application of an infectious virus-like particle system for Marburg virus. J Gen Virol. 2010;91:1325–1334. DOI:10.1099/vir.0.018226-0. 20071483

[26] Mittler E, Kolesnikova L, Hartlieb B, et al. The cytoplasmic domain of Marburg virus GP modulates early steps of viral infection. J Virol. 2011;85:8188–8196. DOI:10.1128/JVI.00453-11. 21680524 PMC3147980

[27] DiCarlo A, Biedenkopf N, Hartlieb B, et al. Phosphorylation of Marburg virus NP region II modulates viral RNA synthesis. J Infect Dis. 2011;204(Suppl 3):S927–S933. DOI:10.1093/infdis/jir319. 21987771

[28] Lipson KL, Ghosh R, Urano F. The role of IRE1alpha in the degradation of insulin mRNA in pancreatic beta-cells. PLoS One. 2008;3:e1648. DOI:10.1371/journal.pone.0001648. 18286202 PMC2241665

[29] Kolesnikova L, Berghöfer B, Bamberg S, et al. Multivesicular bodies as a platform for formation of the Marburg virus envelope. J Virol. 2004;78:12277–12287. DOI:10.1128/JVI.78.22.12277-12287.2004. 15507615 PMC525088

[30] Yoshida H, Haze K, Yanagi H, et al. Identification of the cis-acting endoplasmic reticulum stress response element responsible for transcriptional induction of mammalian glucose-regulated proteins. Involvement of basic leucine zipper transcription factors. J Biol Chem. 1998;273:33741–33749. doi: 10.1074/jbc.273.50.33741 9837962

[31] Iwawaki T, Akai R, Kohno K, et al. A transgenic mouse model for monitoring endoplasmic reticulum stress. Nat Med. 2004;10:98–102. doi: 10.1038/nm970 14702639

[32] Chen X, Shen J, Prywes R. The luminal domain of ATF6 senses endoplasmic reticulum (ER) stress and causes translocation of ATF6 from the ER to the Golgi. J Biol Chem. 2002;277:13045–13052. doi: 10.1074/jbc.M110636200 11821395

[33] Krahling V, Stein DA, Spiegel M, et al. Severe acute respiratory syndrome coronavirus triggers apoptosis via protein kinase R but is resistant to its antiviral activity. J Virol. 2009;83:2298–2309. doi: 10.1128/JVI.01245-08 19109397 PMC2643707

[34] Biedenkopf N, Schlereth J, Grunweller A, et al. RNA binding of Ebola virus VP30 Is essential for activating viral transcription. J Virol. 2016;90:7481–7496. doi: 10.1128/JVI.00271-16 27279615 PMC4984652

[35] Thastrup O, Cullen PJ, Drøbak BK, et al. Thapsigargin, a tumor promoter, discharges intracellular Ca2+ stores by specific inhibition of the endoplasmic reticulum Ca2(+)-ATPase. Proc Natl Acad Sci USA. 1990;87:2466–2470. doi: 10.1073/pnas.87.7.2466 2138778 PMC53710

[36] Criscuolo BA, Krag SS. Selection of tunicamycin-resistant Chinese hamster ovary cells with increased N-acetylglucosaminyltransferase activity. J Cell Biol. 1982;94:586–591. doi: 10.1083/jcb.94.3.586 6215412 PMC2112219

[37] Elbein AD. Inhibitors of the biosynthesis and processing of N-linked oligosaccharides. CRC Crit Rev Biochem. 1984;16:21–49. doi: 10.3109/10409238409102805 6232113

[38] Mittler E, Kolesnikova L, Herwig A, et al. Assembly of the Marburg virus envelope. Cell Microbiol. 2013;15:270–284. doi: 10.1111/cmi.12076 23186212

[39] Li H, Korennykh AV, Behrman SL, et al. Mammalian endoplasmic reticulum stress sensor IRE1 signals by dynamic clustering. Proc Natl Acad Sci USA. 2010;107:16113–16118. doi: 10.1073/pnas.1010580107 20798350 PMC2941319

[40] Perera N, Miller JL, Zitzmann N. The role of the unfolded protein response in dengue virus pathogenesis. Cell Microbiol. 2017;19. doi: 10.1111/cmi.12734 28207988

[41] Brunner JM, Plattet P, Doucey MA, et al. Morbillivirus glycoprotein expression induces ER stress, alters Ca2+ homeostasis and results in the release of vasostatin. PLoS One. 2012;7:e32803. doi: 10.1371/journal.pone.0032803 22403712 PMC3293893

[42] Chan CP, Siu KL, Chin KT, et al. Modulation of the unfolded protein response by the severe acute respiratory syndrome coronavirus spike protein. J Virol. 2006;80:9279–9287. doi: 10.1128/JVI.00659-06 16940539 PMC1563899

[43] Yoshida H, Matsui T, Yamamoto A, et al. XBP1 mRNA is induced by ATF6 and spliced by IRE1 in response to ER stress to produce a highly active transcription factor. Cell. 2001;107:881–891. doi: 10.1016/S0092-8674(01)00611-0 11779464

[44] Mühlberger E, Lötfering B, Klenk HD, et al. Three of the four nucleocapsid proteins of Marburg virus, NP, VP35, and L, are sufficient to mediate replication and transcription of Marburg virus-specific monocistronic minigenomes. J Virol. 1998;72:8756–8764. 9765419 10.1128/jvi.72.11.8756-8764.1998 PMC110291

[45] Enterlein S, Volchkov V, Weik M, et al. Rescue of recombinant Marburg virus from cDNA is dependent on nucleocapsid protein VP30. J Virol. 2006;80:1038–1043. doi: 10.1128/JVI.80.2.1038-1043.2006 16379005 PMC1346851

[46] Fowler T, Bamberg S, Möller P, et al. Inhibition of Marburg virus protein expression and viral release by RNA interference. J Gen Virol. 2005;86:1181–1188. doi: 10.1099/vir.0.80622-0 15784912

[47] Kanda S, Yanagitani K, Yokota Y, et al. Autonomous translational pausing is required for XBP1u mRNA recruitment to the ER via the SRP pathway. Proc Natl Acad Sci USA. 2016;113:E5886–E5895. doi: 10.1073/pnas.1604435113 27651490 PMC5056097

[48] Ambrose RL, Mackenzie JM. ATF6 signaling is required for efficient West Nile virus replication by promoting cell survival and inhibition of innate immune responses. J Virol. 2013;87:2206–2214. doi: 10.1128/JVI.02097-12 23221566 PMC3571479

[49] Zhang P, Su C, Jiang Z, et al. Herpes simplex virus 1 UL41 protein suppresses the IRE1/XBP1 signal pathway of the unfolded protein response via Its RNase activity. J Virol. 2017;91. doi: 10.1128/JVI.02056-16 PMC5286897 27928013

[50] Su A, Wang H, Li Y, et al. Opposite roles of RNase and kinase activities of inositol-requiring enzyme 1 (IRE1) on HSV-1 replication. Viruses. 2017;9. doi: 10.3390/v9090235 PMC5618002 28832521

[51] Stahl S, Burkhart JM, Hinte F, et al. Cytomegalovirus downregulates IRE1 to repress the unfolded protein response. PLoS Pathog. 2013;9:e1003544, doi: 10.1371/journal.ppat.1003544 23950715 PMC3738497

[52] Abdullah A, Ravanan P. The unknown face of IRE1alpha – beyond ER stress. Eur J Cell Biol. 2018;97:359–368. doi: 10.1016/j.ejcb.2018.05.002 29747876

