# Supplementary material for: Marburg virus regulates the IRE1/XBP1-dependent unfolded protein response to ensure efficient viral replication
Source: Emerg Microbes Infect. 2019 Sep 7;8(1):1300–13. doi: 10.1080/22221751.2019.1659552 (PMC6746283; doi:10.1080/22221751.2019.1659552)
Supplement: Supplemental Material [file TEMI_A_1659552_SM8629.pdf]

Supplementary Information

Marburg virus regulates the IRE1/XBP1-dependent unfolded protein response to ensure efficient viral replication

Cornelius Rohde[1,2], Stephan Becker[1,2] and Verena Krähling[1,2,*]

[1]Institut für Virologie, Philipps-Universität Marburg, 35043 Marburg, Hessen, Germany

[2]Deutsches Zentrum für Infektionsforschung (DZIF), Gießen - Marburg - Langen, Marburg, Germany

*Corresponding author:

Verena Krähling, Institut für Virologie, Philipps-Universität Marburg, Hans-Meerwein Str. 2, 35043 Marburg, Hessen, Germany, Phone: +49 6421-2865146, Fax: +49 6421-2868962, E-Mail: kraehliv@staff.uni-marburg.de;

Figure S1. UPRE activation by Tg and Tu in HuH7 cells

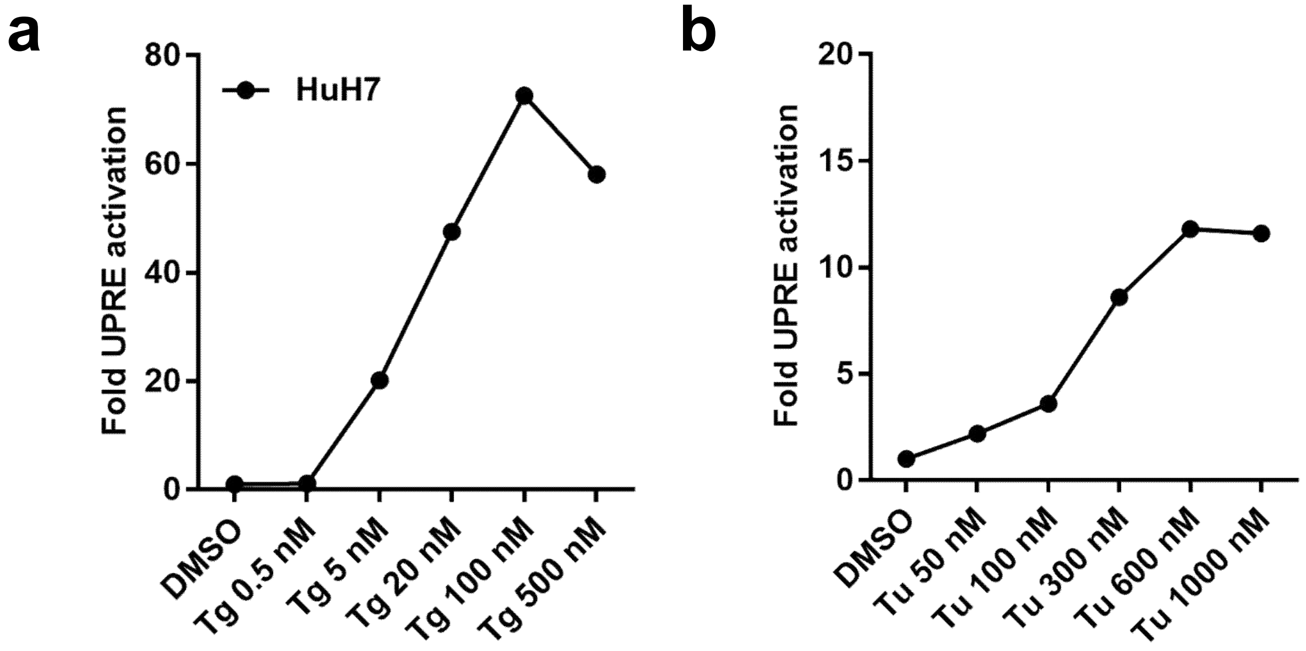

(a, b) HuH7 cells were transfected with plasmids encoding firefly luciferase controlled by a UPRE promoter and with pGL4.73, which constitutively expresses Renilla luciferase. The cells were treated for 16 h with either (a) thapsigargin (Tg) or (b) tunicamycin (Tu) at increasing concentrations to activate the UPRE. Mock-treated cells were treated with vehicle (DMSO). The cells were lysed at 48 h p.t. and luciferase activity was measured. Firefly luciferase activity was normalized to Renilla activity; the fold induction compared to DMSO-treated cells (set to 1) was calculated and is shown.

Figure S2. UPRE activation by GP deletion mutants

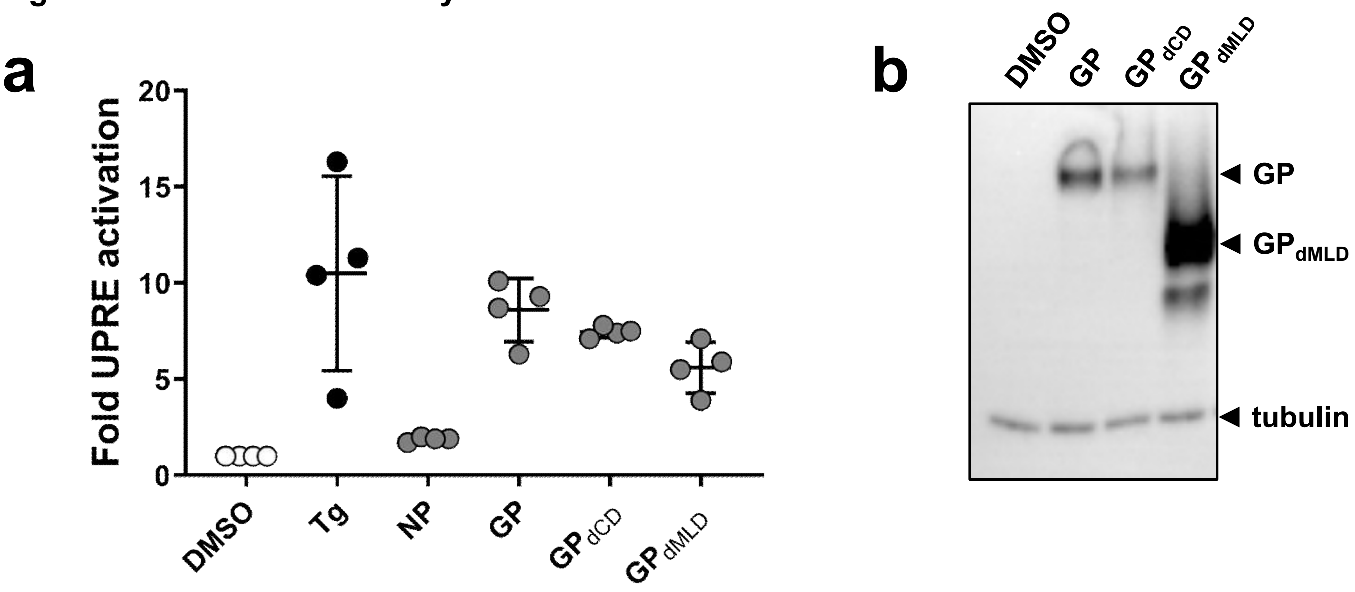

(a, b) HuH7 cells were transfected with plasmids encoding firefly luciferase controlled by a UPRE promoter (1 µg) and with pGL4.73 (0.1 µg), which encodes Renilla luciferase, and with plasmids encoding NP, GP and deletion mutants of GP (1 µg each). A truncated GP protein lacking either the mucin-like domain (dMLD) or the cytoplasmic domain of eight amino acids (dCD) were used. HuH7 cells transfected with the empty vector were treated with vehicle (DMSO) or with Tg (5 nM). The cells were lysed at 48 h p.t. and analyzed using luciferase assays. Firefly luciferase activity was normalized to Renilla activity, and the fold induction in comparison to the DMSO control (set to 1) was calculated. The experiment was performed four times. **(b)** Equal amounts of the cell lysates were subjected to Western blotting using monoclonal antibodies against GP and tubulin. The experiment was performed four times; the results of one representative experiment are shown.

Figure S3. Localization pattern of GP and GP_{dMLD}

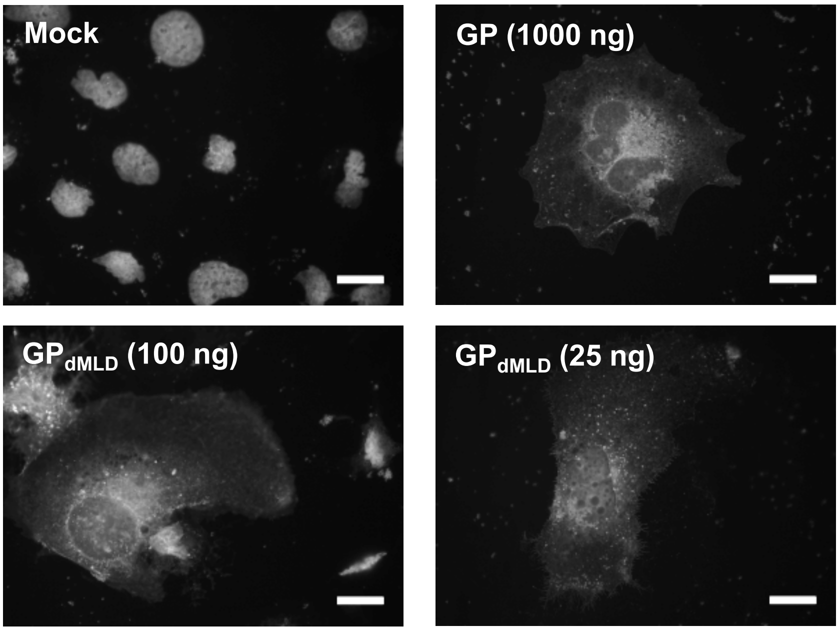

Immunofluorescence analysis of HuH7 cells transfected with plasmids encoding GP or GP_{dMLD}. Cells were fixed at 48 h p.t. and stained using a mouse monoclonal antibody against GP. Cell nuclei of Mock cells were visualized by DAPI staining. Scale bar = 25 µm.

Figure S4. XBP1s-GFP expressing cells

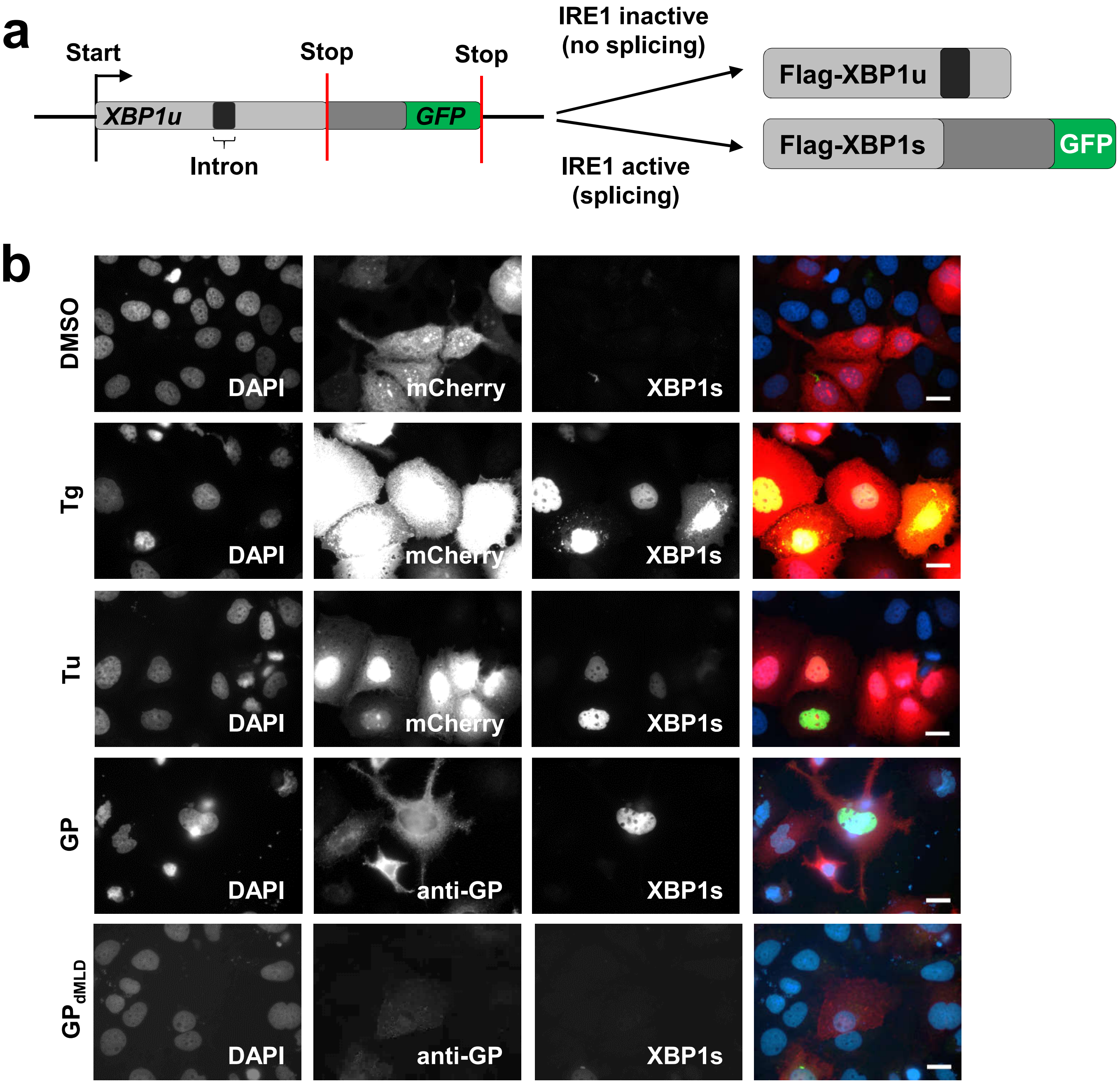

(a) Schematic overview of the XBP1-GFP expression plasmid. *XBP1-GFP* mRNA can be translated into the XBP1u or the XBP1s-GFP protein dependent on IRE1-mediated splicing. Both proteins carry a N-terminal Flag-tag. **(b)** Exemplary photomicrographs. Percentage of XBP1s-GFP positive nuclei were counted in cells expressing either the viral protein or mCherry (DMSO and Tg) to obtain the results shown in Fig. 2C. Scale bars = 25 µm.

Figure S5. UPRE activation by Tg and Tu in HAP1 cells

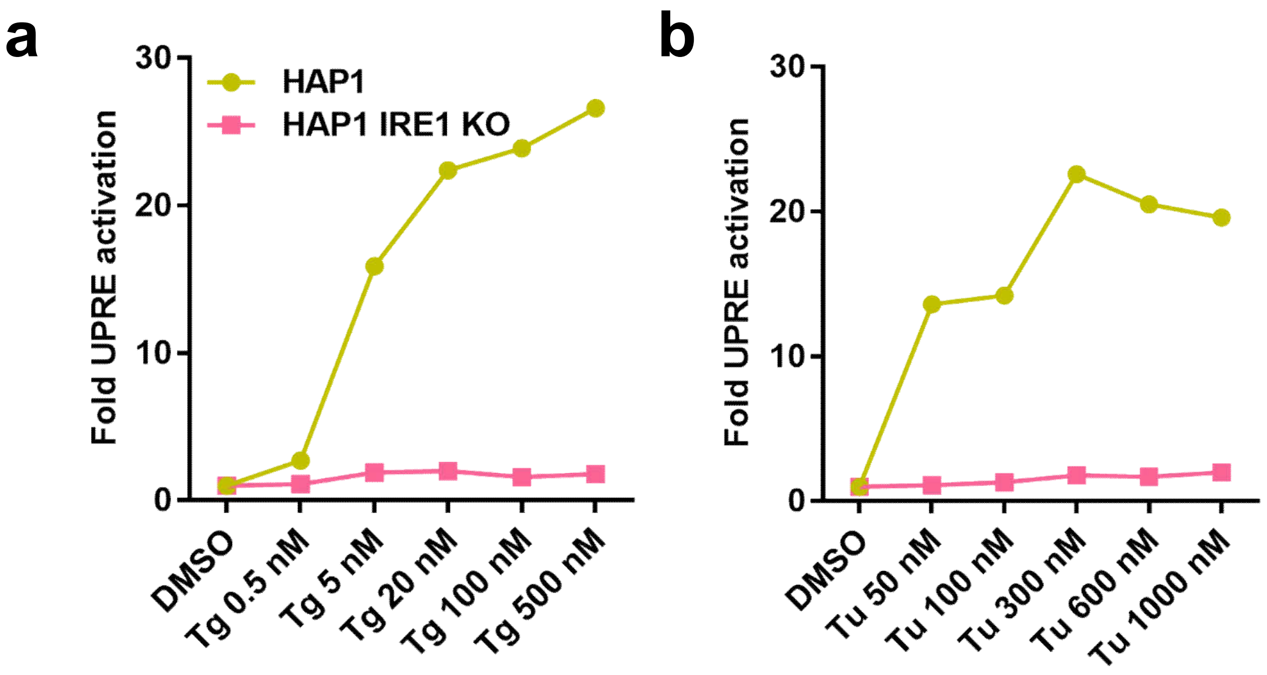

(**a, b**) HAP1 and HAP1 IRE1 KO cells were transfected, treated and harvested as described in the legend to Figure S1.

Figure S6. VP30 interacts with XBP1u, but not with XBP1s-GFP or IRE1

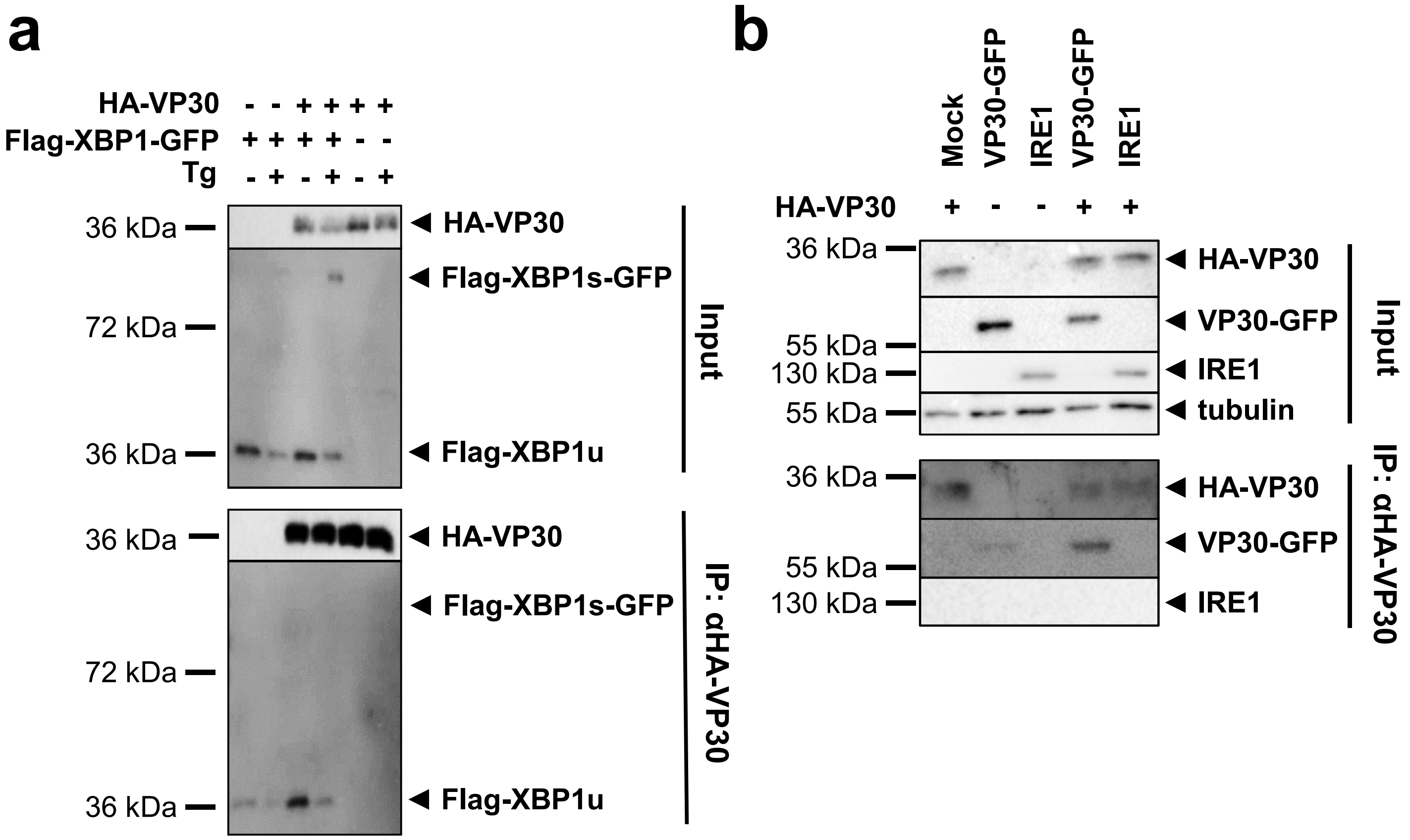

(a, b) HuH7 cells were transfected and harvested as described in the legend to Figure 5 with HA-VP30, VP30-GFP, XBP1, IRE1 expression plasmids or empty vector (pCAGGS). The experiment was repeated three times and one exemplary Western blot is shown. **(a)** To induce the expression of XBP1s, the cells were treated 32 h p.t. for 16 h with Tg (5 nM) or the vehicle control DMSO.

Figure S7. MARV infection of HuH7 cells (from Fig. 6)

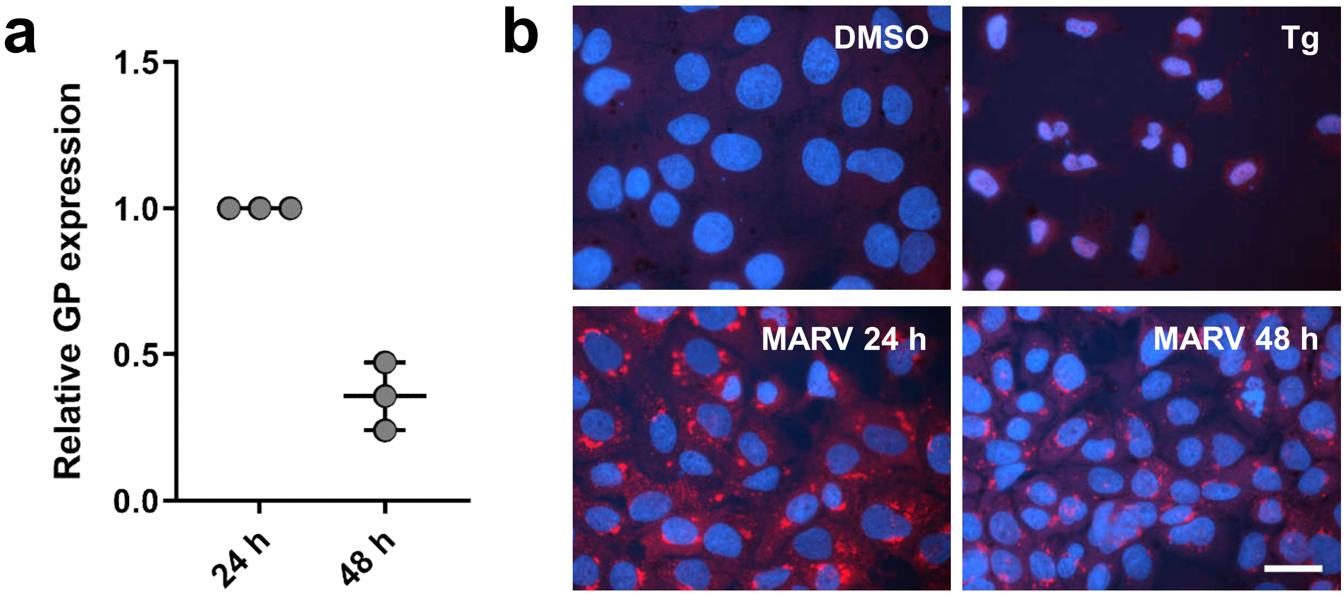

(a) GP protein amounts detected in infected cells by Western blot (from Fig. 6a, 6b) were quantified. Tubulin levels were used to normalize the amount, GP amount at 24 h p.i. was set to 1; data are shown as the means ± SD. **(b)** HuH7 cells from Fig. 6 were analysed by IFA to show that all cells were infected at 24 p.i. with MARV. The nucleoprotein of the virus was detected using a monoclonal antibody and a secondary antibody conjugated to Alexa594. DAPI staining was used to visualize the cell nuclei. Exemplary photomicrographs are shown. Scale bar = 25 µm.
